# Relation between endoleak development and aneurysm diameter in patients with abdominal aorta aneurysm treated with endovascular repair

**DOI:** 10.1186/1749-8090-10-S1-A224

**Published:** 2015-12-16

**Authors:** Serkan Yazman, İsmail Yürekli, Ufuk Yetkin, Levent Yılık, Köksal Dönmez, Hasan İner, Tevfik Güneş, Barçın Özcem, Ali Gürbüz

**Affiliations:** 1 Department of Cardiovascular Surgery, Katip Celebi University Izmir Ataturk Training and Research Hospital, Izmir, Turkey

## Background/Introduction

Although it may differ with age, gender and body mass index; diameter of infrarenal aorta is between 1 and 2,5 cm. Its mean value in male is 21,4 mm and 18,7 mm in female. More than 50% irreversible dilation of abdominal aorta is called abdominal aorta aneurysm. It is usually observed as dilation of aortic diameter over 3 cm between aortic bifurcation and renal artery.

## Aims/Objectives

We retrospectively investigated and included elective or emergency (ruptured abdominal aortic aneurysm) intervention of 203 abdominal aortic aneurysm patients treated with endovascular aortic repair (EVAR) between dates January 2006 and January 2013. Sixteen of these patients were female (7,9%) and 187 of them were male (92,1%).

## Method

In our study mean aneurysm diameter was 65,83 mm ± 14,92 mm (between 40 mm and 130 mm).

## Results

When we compared endoleak and aneurysm diameter; mean diameter was 64,7 ± 16,96 mm in endoleak group and 68,22 ± 14,61 mm in group without endoleak. There was not any statistical significance.

## Discussion/Conclusion

Intervention is indicated in patients with abdominal aorta aneurysm diameter over 5 cm, symptomatic patients with aneurysm diameter less than 5 cm, equal to or more than 5 mm dilation of aneurysm in six months, ruptured or prone to rupture aneurysms, saccular or dissected aneurysms.

## Consent

Written informed consent was obtained from the patient for publication of this Case report and any accompanying images. A copy of the written consent is available for review by the Editor-in-Chief of this journal.

